# Coronary Artery Calcium Score as a Predictor of Cardiovascular Disease Events and Mortality Among Asymptomatic Working-Age Adults: A Systematic Review

**DOI:** 10.7759/cureus.101388

**Published:** 2026-01-12

**Authors:** Marinos Charalambous, Robert Filler, Elpidoforos S Soteriades, Richard Abitria, Jeff Kiser, Alejandro Fernandez-Montero, Stefanos N Kales, Denise Smith

**Affiliations:** 1 Department of Basic and Clinical Sciences, University of Nicosia Medical School, Nicosia, CYP; 2 Environmental and Occupational Medicine and Epidemiology, Department of Environmental Health, Harvard T.H. Chan School of Public Health, Boston, USA; 3 School of Economics and Management, Healthcare Management Program, Open University of Cyprus, Nicosia, CYP; 4 78th Occupational Medicine Services, Robins Air Force Base, Warner Robins, USA; 5 First Responder Health and Safety Lab, Department of Health and Human Physiological Sciences, Skidmore College, Saratoga Springs, USA

**Keywords:** calcium, cardiac events, cardiovascular diseases, coronary artery disease, mortality, predictor, prognosis

## Abstract

The Coronary Artery Calcium (CAC) score is primarily used in asymptomatic individuals at intermediate risk for developing cardiovascular disease (CVD), particularly when there is uncertainty whether to initiate statins or intensify primary prevention measures. In this systematic review, we examined the value of the CAC score as a predictor of incident CVD events and mortality among asymptomatic working-age adults. We reviewed studies published from November 2009 to December 2022 examining asymptomatic adults 18-65 years of age. A total of 908 studies were identified. After the elimination of 578 and 326 studies based on abstract and full-text review, respectively, we examined four studies that met the inclusion criteria. Two of the studies showed that any CAC score above zero was associated with a four- to fivefold increased risk of incident cardiac events. In addition, we found that increasing CAC score was associated with a higher risk of incident cardiac events and/or all-cause mortality in a dose-response relationship documenting a strong criterion of causality. Furthermore, one of the studies elicited a higher discriminatory power of the CAC score compared to the Framingham and the Atherosclerotic Cardiovascular Disease (ASCVD) risk scores in distinguishing high-risk from intermediate-risk individuals for all-cause mortality. Our review showed that the CAC score is predictive of elevated risk for incident CVD events and mortality among asymptomatic working-age adults. However, prospective studies are warranted to perform cost-benefit analyses on the utility of CAC scoring as a screening tool in the general population.

## Introduction and background

Cardiovascular disease (CVD) remains a leading cause of death worldwide and the most common cause of death among adults in the United States [1]. In the United States, one in three deaths is attributable to CVD [1], resulting in enormous personal loss and tremendous socioeconomic consequences, including a cost of more than $200 billion annually [2]. Heart disease is responsible for the highest morbidity, mortality, and associated societal costs compared to any other disease or injury among adults of working age [3]. Death before retirement is commonly attributed to CVD, accounting for 36% and 27% of premature deaths in men and women, respectively. Coronary heart disease (CHD) is responsible for approximately one in four (23%) deaths before the age of 65 years in men and one out of eight deaths (13%) in women of the same age group [3].

Therefore, focusing on CVD prevention is of utmost importance. Prevention of CVD is guided by risk stratification, which is currently based on several CVD risk assessment models [1]. Enhancing current CVD risk assessment could improve CVD prevention efforts and reduce associated premature morbidity and mortality among asymptomatic working-age individuals.

Many CVD risk assessment models are available for use in apparently healthy, asymptomatic individuals. The Framingham Heart Study was the first to initiate the concept of CVD risk assessment and primary prevention of CVD since the recruitment of the first study participants in 1948 [4,5]. The American Heart Association and American College of Cardiology 2013 guidelines on the assessment of cardiovascular risk introduced the pooled cohort equations, which are now widely used [6]. Furthermore, the authors of the European guidelines on CVD prevention in clinical practice [7] have recommended the use of the Systematic Coronary Risk Evaluation (SCORE) system since 2003. Other risk assessment models include the ASSIGN (CV risk estimation model from the Scottish Intercollegiate Guidelines Network) [8], the Q-Risk [9,10], the Prospective Cardiovascular Münster (PROCAM) study [11], the CUORE [12], the Arriba [13], the Globorisk [14], and the Reynolds risk score for men and women [15,16]. Overall, the various risk assessment models perform similarly when applied to populations comparable to those from which they were derived. Nevertheless, risk assessment models have limitations that may lead to under- or over-estimation of risk in specific populations, which may include certain ethnic groups, younger individuals, and women [17-19]. For example, the PROCAM was developed from a male-dominated workforce; the ASSIGN, SCORE, and Globorisk were based mainly on European populations; and CUORE has limited applicability for populations outside of Italy. In addition, the Reynolds risk score was developed using two specific cohorts of healthy US health professionals (the Women's Health Study and the Physicians' Health Study) and has limited generalizability and diversity.

Adding the Coronary Artery Calcium (CAC) score to existing CVD risk assessment models has been demonstrated to significantly boost the C-statistic (a measure of discrimination) by roughly 0.029 for overall CVD and up to 0.049 for coronary heart disease (CHD) risk, showing better distinction between event-goers and non-event-goers [20]. Furthermore, reclassification (correctly moving people into higher or lower risk groups) is also improved, especially for intermediate-risk individuals (typically 5%-20% 10-year risk). Reclassifying intermediate (defined as 10%-20% and 6%-20%) risk subjects with CAC < 100 to the low-risk category and with CAC ≥ 400 to the high-risk category yields a net reclassification index (NRI) of 21.7% (p = 0.0002) and 30.6% (p < 0.0001) for the Framingham risk score (FRS), respectively [21]. In this group, the NRI can reach 46%-66%, effectively identifying those who truly need statins or aspirin and those who do not.

Nevertheless, the use of CAC scoring has not been established as a commonplace practice for screening in the general adult population. Currently, CAC scoring is primarily used in the assessment of selected individuals who have intermediate CVD risk based on the presence of traditional CVD risk factors [22-24]. The CAC score has been viewed as particularly useful when there is uncertainty about whether to start a statin and intensify primary prevention measures [23]. Although radiation exposure to obtain a CAC score has been substantially reduced in recent years and the technique is now readily available at a modest cost in most communities, the role of the CAC score as a routine screening tool for asymptomatic individuals has not been established due to concerns over cost-effectiveness or lack of consensus. Major guidelines still position it primarily for refining risk in intermediate-risk, asymptomatic individuals for shared decision-making, but not as a universal routine screening tool [22]. Assessing the ability of the CAC score to predict CVD events and mortality is a necessary step in estimating its cost-effectiveness for this purpose.

The 2018 US Preventive Services Task Force (USPSTF) recommendations on the use of CAC scoring suggested that, in asymptomatic adults, the evidence was insufficient to assess the balance of benefits and harms with respect to the use of the CAC score for reducing incident CVD events or mortality. While CAC scores improve risk prediction, the clinical significance of these improvements and whether they lead to better treatment decisions and outcomes (such as fewer heart attacks) was not proven by trials, with potential harms such as radiation exposure and cost of over-testing [1]. However, the American Heart Association/American College of Cardiology [23], Society of Cardiovascular Computed Tomography [24], and European guidelines on CVD prevention [25] provide recommendations in favor of the CAC score being used to aid in the risk assessment of selected individuals. However, further evidence is needed to develop decision-making algorithms for CAC score utilization in asymptomatic individuals or defined sub-populations, including age- and gender-specific subsets who may benefit from enhanced CVD risk assessment with CAC scoring.

The main objective of our systematic review was to assess the existing literature and examine the value of the CAC score as a predictor of incident CVD events and mortality among asymptomatic working-age adults. We focused on this subset of the population because of their unique risks and potential benefits from improved risk stratification and the inconclusive nature of existing recommendations regarding the use of CAC scoring for reducing incident CVD events or mortality in asymptomatic adults, as well as the profound toll of CVD among working-age adults [3]. This subset of the population may benefit the most from early and improved screening, considering their younger age distribution.

## Review

Our review protocol was designed to identify and include studies examining asymptomatic adults between the ages of 18 and 65 years old and was registered in PROSPERO in April 2020 (registration ID: CRD42020167227). This study, as a literature review, was exempt from institutional review board and ethics committee approval.

**Figure 1:**
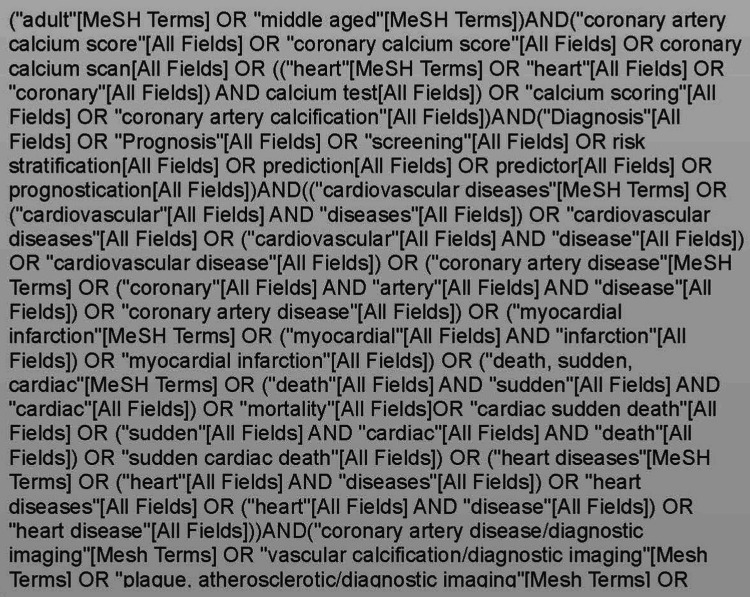
Search strategy comprising key terms and MeSH MeSH: Medical Subject Headings

Eligible articles included cross-sectional, cohort, and case-control studies, while case reports and reviews were excluded. For studies where age ranges were not reported, inclusion was allowed if the mean age was less than 3 standard deviations below 65 years. Studies with data stratified by age were included, provided that the stratified data otherwise met the inclusion criteria. Studies of participants with preexisting coronary artery disease (CAD) or other high-risk conditions (familial hypercholesterolemia, diabetes mellitus, rheumatoid arthritis, or patients on dialysis) were excluded. We also excluded studies where the CAC score was not used as an independent exposure variable, and a subsequent CVD event was not the outcome of interest. The initial literature review was implemented by searching PubMed for articles published between November 2009 and December 2022 using a broad search strategy comprising key terms and Medical Subject Headings (MeSH) (Figure 1).

The bibliographies of the review articles captured within our search strategy were manually searched to identify additional articles not captured in our digital search, as well as references to relevant datasets of the search. A detailed literature search was conducted by two primary reviewers (MC and RF) based on independent reviews of titles and abstracts. Both researchers were tasked with independently identifying studies, extracting the data, verifying complete data collection, and grading the quality of the results. Subsequently, a full-text review of 330 articles that met the initial eligibility criteria was performed by MC, RF, and RA using a spreadsheet to uniformly capture data on study design, participants, exposures, datasets, and outcomes from the studies reviewed. The standard data collection tool allowed for methodical review of many publications originating from a smaller number of datasets. Another reviewer (SK) was asked to adjudicate and resolve any disagreements between the independent reviewers.

Thereafter, the full-text articles were assessed independently by the reviewers using our predefined criteria. When screening the studies, three categories were used for articles: included, excluded, or under consideration. Any uncertainties regarding study inclusion were discussed between the two reviewers. In the event of disagreement, a discussion was held with the third reviewer, and disagreements were resolved by the third reviewer. We extracted the data into a standard spreadsheet for use as the data collection tool. The relevant data included the study dataset, number of enrolled participants, study design, duration of follow-up, age range of participants, and studied outcomes.

**Figure 2:**
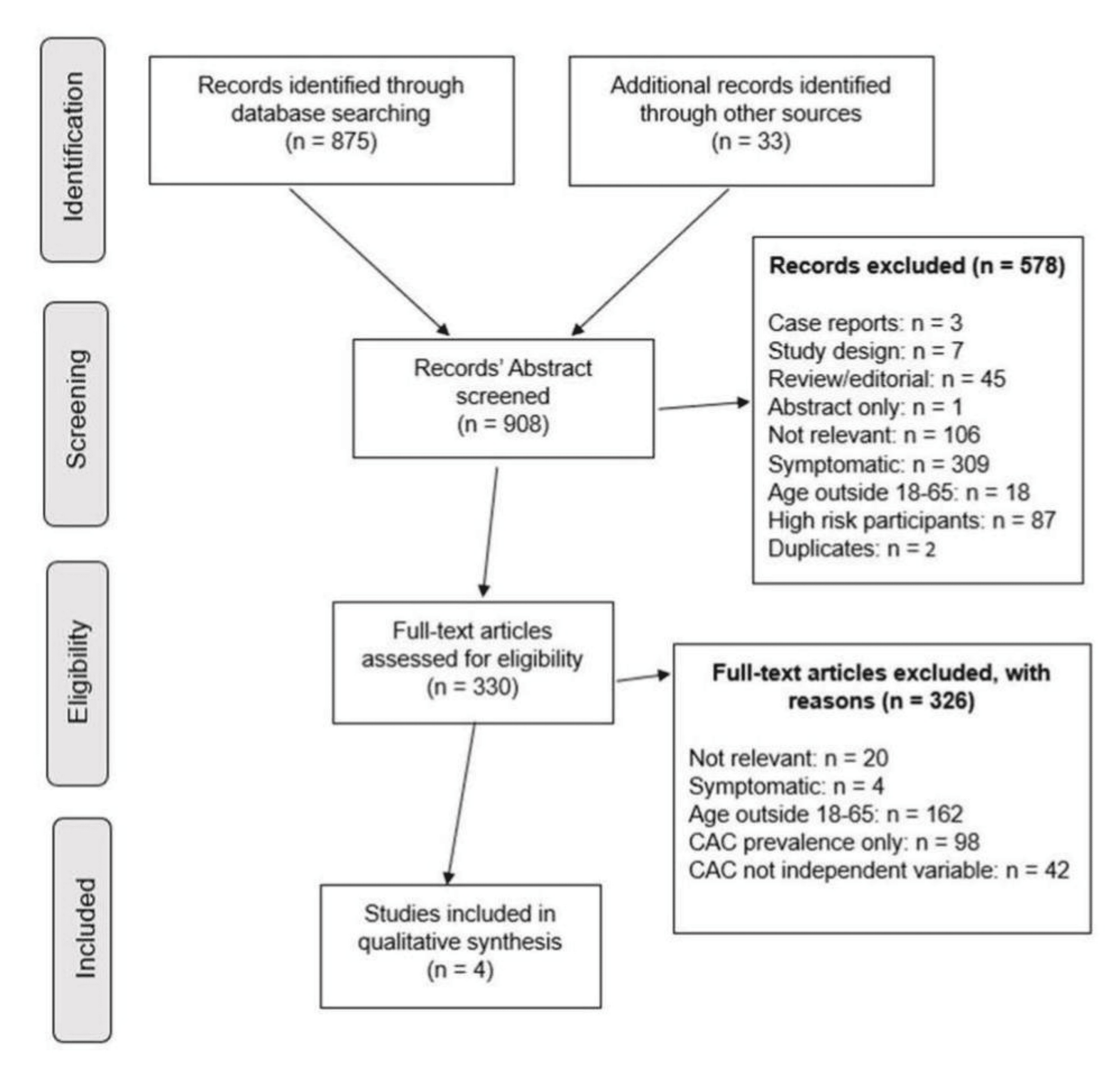
Study selection process CAC: Coronary Artery Calcium

Using the initial search terms, we identified a total of 908 studies. Following abstract screening, we eliminated 578 studies, as shown in Figure 2. Based on an exhaustive full-text review of the remaining articles (a total of 330 articles), we were able to identify only four articles that met the inclusion criteria, focusing on asymptomatic adults of working age (Figure 2). Due to the small number of studies included in our final selection, we were not able to perform a quantitative meta-analysis. A total of 162 otherwise eligible studies included participants with ages outside of the designated 18-65 age range. Additionally, 98 studies retrieved by the search protocol only measured CAC prevalence, and another 42 studies did not use the CAC score as the independent variable.

The main findings of the four studies included in our systematic review are summarized in Table 1.

**Table 1:** Summary of outcome measures in the selected articles included in the final systematic review CI: confidence interval, CAC: Coronary Artery Calcium, ACM: all-cause mortality, CHD: coronary heart disease

Study article (publication year)	End points	Hazard ratios (95% CI) for CAC scores				
Jin et al. (2012) [26]	Cardiac events	Any CAC > 0				4.3 (1.9-9.7)
Carr et al. (2017) [27]	-	CAC: 0	CAC: 1-99	CAC:100-399	CAC: ≥400	
	ACM men < 45	1.0 (ref)	3.1 (0.8-12.7)	3.3 (0.0-53.4)	4.9 (0.5-51.0)	
	ACM women < 55	1.0 (ref)	0.3 (0.04-2.8)	2.5 (0.3-22.3)	4.7 (0.4-55.5)	
	ACM men 45-74	1.0 (ref)	1.8 (1.1-2.8)	2.5 (1.5-4.0)	4.5 (2.8-7.1)	
	ACM women 55-74	1.0 (ref)	2.4 (1.2-4.8)	3.8 (1.8-7.9)	5.8 (2.8-12.4)	
	ACM men ≥ 75	1.0 (ref)	1.3 (0.6-2.9)	0.9 (0.4-2.0)	1.5 (0.7-3.1)	
	ACM women ≥ 75	1.0 (ref)	1.5 (0.5-4.4)	3.1 (1.1-8.7)	2.7 (0.99-7.5)	
	Entire study population	1.0 (ref)	1.5 (1.1-2.1)	1.8 (1.3-2.5)	2.6 (1.69-3.6)	
Nakanishi et al. (2016) [28]	-	CAC: 0	CAC: 1-19	CAC: 20-99	CAC: ≥100	Any CAC > 0
	CHD events	1.0 (ref)	2.6 (1.0-5.7)	5.8 (2.6-12.1)	9.8 (4.5-20.5)	5.0 (2.8-8.7)
	CVD events	-	-	-	-	3.0 (1.9-4.7)
	ACM	1.0 (ref)	1.1 (0.5-2.1)	0.9 (0.3-2.7)	3.7 (1.5-10.0)	1.6 (1.0-2.6)
Han et al. (2020) [29]	ACM event rate	Low-risk group (as per CAC): 0.9 per 1,000 person-years, intermediate-risk group (as per CAC): 2 per 1,000 person-years, high-risk group (as per CAC): 5.2 per 1,000 person-years				

Coronary heart disease (CHD) events included fatal or nonfatal myocardial infarction, acute coronary syndrome without myocardial infarction, coronary revascularization, or CAD death. Incident cardiovascular disease (CVD) events included CHD, stroke, heart failure, and peripheral arterial disease.

Subclinical coronary atherosclerosis in young adults: prevalence, characteristics, predictors with coronary computed tomography angiography

In this study, Jin et al. attempted to assess predictors of subclinical coronary atherosclerosis and cardiac events among young adults. This study involved 914 asymptomatic individuals, under the age of 45 years, who underwent both coronary computed tomography (CT) angiography and CAC scoring between January 2006 and December 2008, at Seoul National University Bundang Hospital. Successful follow-up occurred in 100% of the subjects, and the mean follow-up duration was 26.8 ± 14.6 months. Subclinical coronary atherosclerosis was found in 86 subjects (9.4%). Notably, the most common type of plaque was noncalcified plaque and was found in 63 subjects (6.9% out of 9.4%). Cardiac events included cardiac death, nonfatal myocardial infarction, and unstable angina requiring hospitalization and/or revascularization later than 90 days following coronary CT. The study reported 2.45 cardiac events per 1,000 person-years of follow-up and demonstrated that individuals with the presence of any CAC score greater than zero had a hazard ratio (HR) for cardiac events of 4.35 (1.95-9.72), p < 0.0001 (Table 1) [26].

Association of Coronary Artery Calcium in adults aged 32 to 46 years with incident coronary heart disease and death

The Coronary Artery Risk Development in Young Adults (CARDIA) study is a prospective community-based study that recruited 5,115 black and white participants aged 18-30 years from March 25, 1985, to June 7, 1986. The cohort has been under surveillance for 30 years, with CAC measured at 15 (n = 3043), 20 (n = 3141), and 25 (n = 3,189) years after recruitment. The mean follow-up period for incident events was 12.5 years. Incident coronary heart disease (CHD) events included fatal or nonfatal myocardial infarction, acute coronary syndrome with or without myocardial infarction, coronary revascularization, or CAD death. Incident CVD events included CHD, stroke, heart failure, and peripheral arterial disease. Death included all causes. During follow-up, there were 165 incident CVD events, including 57 incident CHD events. After adjusting for demographics and risk factors, those with any CAC score experienced a fivefold increase in CHD events (HR: 5.0; 95% confidence interval (CI): 2.8-8.7) and a threefold increase in CVD events (HR: 3.0; 95% CI: 1.9-4.7) compared to those participants with a CAC score of zero. Within CAC score strata of 1-19, 20-99, and 100 or more, the HRs for CHD were 2.6 (95% CI: 1.0-5.7), 5.8 (95% CI: 2.6-12.1), and 9.8 (95% CI: 4.5-20.5), respectively. A CAC score of 100 or more had an incidence density of 22.4 deaths per 100 participants followed up for 12.5 years (HR: 3.7; 95% CI: 1.5-10.0); of the 13 deaths in participants with a CAC score of 100 or more, 10 were adjudicated as CHD events. Compared with no CAC, the adjusted hazard ratio for CHD among those with any CAC above zero was 5.0 (95% CI: 2.8-8.7; p < 0.001). The incidence density of any CHD event increased from 4.8 events per 100 persons at a CAC score of 1-19 (HR: 2.6; 95% CI: 1.0-5.7; p = 0.03) to 10.6 events per 100 persons at a CAC score of 20-99 (HR: 5.8; 95% CI: 2.6-12.1; p < 0.001) and to 26.1 events per 100 persons at a CAC score of 100 or more (HR: 9.8; 95% CI: 4.5-20.5; p < 0.001). In addition, a CAC score of 100 or more was associated with premature death. The presence of any CAC score above zero had an incidence density of 8.1 for all-cause mortality per 100 people followed up for 12.5 years (HR: 1.6; 95% CI: 1.0-2.6; p = 0.05). The incidence density increased to 22.4 deaths per 100 people in those with a CAC score of 100 or more (HR: 3.7; 95% CI: 1.5-10.0; p < 0.001) [27].

All-cause mortality by age and gender based on Coronary Artery Calcium scores

In this study, Nakanishi et al. assessed the long-term predictive value of the CAC score for all-cause mortality. This study involved 13,092 asymptomatic participants with no known coronary artery disease at baseline who underwent a CAC scan between July 1997 and December 2011, at the Los Angeles Biomedical Institute at Harbor University of California, Los Angeles (UCLA) Medical Center. The participants were assigned to CAC categories 0, 1-99, 100-399, and ≥400. Multivariable Cox proportional hazard models were used to calculate adjusted HRs for all-cause mortality, stratified by gender and age (younger, intermediate, or older group). The younger age group was designated at <45 years for men and <55 years for women. The mean age for the younger groups were 39.9 and 47.5 years for men and women, respectively. During a 15-year follow-up, the mortality rates of those with CAC = 0 were very low at 0.4 and 0.9 per 1,000 person-years of follow-up in younger men and women, respectively. Patients with CAC = 0 had a lower rate of mortality at all ages compared with the general United States population (2012). Compared with CAC = 0 (used as reference), the higher the CAC score category, the higher the trend for all-cause mortality (Table 1). This observation was true for the entire study population; however, in the age-stratified calculation of HRs, this observation did not achieve statistical significance for the younger and older age groups [28].

Machine learning based risk prediction model for asymptomatic individuals who underwent Coronary Artery Calcium score: comparison with traditional risk prediction approaches

This study included a total of 86,155 asymptomatic individuals from South Korea who underwent CAC scoring between December 2002 and July 2014 as part of a health checkup. The study was part of the Korea Initiative Coronary Calcification (KOICA) registry. The participants were followed for a median duration of 4.6 years (interquartile range (IQR): 3.0-6.6 years). A machine learning (ML)-based risk-prediction model was developed, and its predictive value for all-cause mortality was compared to conventional CVD risk prediction algorithms. The machine learning algorithm included 70 parameters, which consisted of 35 clinical, 32 laboratory, and 3 CAC score parameters. A separate cohort was used to externally validate the developed machine learning (ML) algorithm. The performance of the ML algorithm for predicting all-cause mortality was compared to the following models: (i) the Framingham risk score (FRS) + CACs, (ii) atherosclerotic cardiovascular disease (ASCVD) risk score + CACs, and (iii) a logistic regression (LR) model. The study participants were categorized into low-, intermediate-, or high-risk groups, based on the machine learning risk prediction algorithm. More than half of the participants were classified as the low-risk group (60%). About a third of the participants (30%) were classified as the intermediate-risk group, and 10% as the high-risk group. During a median of 4.6 years of follow-up (IQR: 3.0-6.6 years), 690 (0.8%) all-cause fatality events occurred. The incidence of all-cause mortality per 1,000 person-years was 0.4 (95% confidence interval (CI): 0.3-0.6), 1.5 (95% CI: 1.2-1.9), and 5.7 (95% CI: 4.6-6.9) in the low-, intermediate-, and high-risk groups, respectively. The machine learning-based algorithm performed better in classifying patients to low-, intermediate-, or high-risk groups compared to the Framingham risk score, the ASCVD risk score, or the CAC score alone. This was evident by the finding that the lowest and highest incidence for all-cause mortality per 1,000 person-years were observed in the groups identified as low- and high-risk by the machine learning algorithm, respectively [29].

The CAC score was as good as the Framingham risk score in classifying patients as low risk for all-cause mortality but better than the Framingham risk score in distinguishing high-risk from intermediate-risk patients. In addition, the CAC score was better than the ASCVD risk score in identifying patients who had a higher risk for all-cause mortality.

To date, CAC scoring has been recommended primarily as an assessment tool for individuals who have intermediate CVD risk based on the presence of traditional CVD risk factors in order to guide decisions about treatment [23,24]. The CAC score has been suggested as particularly useful when there is uncertainty whether to initiate a statin or intensify primary prevention measures [23]. Assessing the CAC score has been previously reported to improve medication adherence, as well as behavioral modification and lifestyle changes that are beneficial for lowering CAD risk [30]. However, the potential role of the CAC score as a widespread screening tool among asymptomatic individuals has not been well studied. An important step in this direction is to determine the ability of the CAC score to predict CVD events and mortality in the general population and consequently estimate its cost-effectiveness.

Our review focused on the recent literature regarding the utility of the CAC score in predicting incident CVD events and mortality among asymptomatic working-age adults. In summary, our systematic review showed that the CAC score can predict an elevated risk of incident CVD events and mortality among asymptomatic working-age adults. Specifically, two out of the four identified studies showed that any CAC score above zero was associated with a four- to fivefold increased risk of incident CVD events. In addition, two of the studies showed that increasing CAC score categories were associated with a higher risk of incident CHD events and/or all-cause mortality in a dose-response relationship, documenting a strong criterion of causality. All included studies showed significant findings for both CVD events and mortality. Furthermore, one of the studies, with the help of a machine learning tool, elicited a higher discriminatory power of the CAC score (compared to the Framingham and ASCVD risk scores) for distinguishing high-risk from intermediate-risk individuals for all-cause mortality.

In 2018, the US Preventive Services Task Force report suggested that the evidence was insufficient to assess the balance of benefits and harms with respect to the use of the CAC score in asymptomatic adults for reducing incident CVD events or mortality [1]. However, our systematic review identified articles [26-29] that documented an elevated risk for cardiac events and mortality among asymptomatic adults with a CAC score higher than zero. Three out of four articles included in our systematic review had been published between the years 2012 and 2017 [26-28]. Notably, none of the studies included in our systematic review were referenced in the 2018 USPSTF report.

All four studies [26-29] included in our review showed statistically significant findings suggesting that CAC scoring may play an important role in CVD risk stratification of asymptomatic individuals for predicting incident CVD events and mortality. The study by Jin et al. (article 1) was the first to demonstrate an increased risk of cardiac events (cardiac death, nonfatal myocardial infarction, and unstable angina requiring hospitalization or revascularization later than 90 days following coronary CT) in asymptomatic individuals with any CAC score greater than zero [26]. The study included 914 asymptomatic individuals under the age of 45 who had undergone both coronary CT angiography and CAC scoring and had a mean follow-up duration of 26.8 ± 14.6 months. This study showed that individuals with any CAC score greater than zero had an annual risk of cardiac events that was more than four times higher compared to participants with a zero calcium score. The CT angiography part of the study showed that the prevalence of subclinical coronary atherosclerosis among asymptomatic young adults is not negligible (9.4%) and that most of those study participants with subclinical atherosclerosis had noncalcified plaques (6.9% out of 9.4%). This finding is important since it highlights a limitation of CAC scoring among adults younger than 45 years. Subclinical atherosclerosis may be present in the form of noncalcified plaques despite a calcium score of zero, and computed tomography (CT) coronary angiography is essential in identifying such noncalcified plaques. Thus, a CAC score greater than zero is associated with greater risk, but a zero CAC score does not assure that there is no subclinical disease.

Furthermore, Carr et al. (article 2) reported on the Coronary Artery Risk Development in Young Adults (CARDIA) study, which was a prospective community-based study that recruited 5,115 black and white participants aged 18-30 years with a mean follow-up period for incident cardiac events of 12.5 years [27]. After adjusting for demographics and risk factors, those with any CAC experienced a fivefold increase in CHD events and a threefold increase in CVD events compared to those with a CAC score of zero. In addition, a CAC score of 100 or more was associated with a higher risk of premature death. This study highlights the important value of CAC screening among asymptomatic individuals younger than 50 years of age. The ability of CAC scoring to identify asymptomatic individuals at increased risk is evident, and what remains to be investigated is its cost-effectiveness.

Nakanishi et al. (article 3) were the first study to demonstrate the predictive utility of the CAC score in asymptomatic individuals stratified by age and gender [28]. The study included 13,092 asymptomatic patients without known CVD who underwent CAC scoring and had a median follow-up of at least 10 years. The younger age group was designated at <45 years for men and <55 years for women. The study showed a trend toward higher mortality with increasing CAC score in young- and middle-aged men and women. In older patients, the value of the CAC score for long-term risk stratification was lower due to competing risks associated with an increased overall mortality in older patients. However, even in older patients, those with zero or low CAC scores were at a significantly lower risk of mortality compared to the general population. It is important to note that a CAC score of zero was previously shown to confer an extremely low mortality risk, over an average of 5.6 years of follow-up, in patients who were referred for calcium scoring for specific reasons [31] and in participants of the Multi-Ethnic Study of Atherosclerosis (MESA) study who were asymptomatic men and women aged 45-84 years [32].

Finally, the study by Han et al. (article 4) included a total of 86,155 asymptomatic individuals from South Korea who underwent CAC scoring as part of a health checkup [29]. A machine learning-based risk prediction model was developed, and its ability to predict all-cause mortality was compared to that of conventional CVD risk prediction algorithms (i.e., the ASCVD risk score and the Framingham risk score) and the CAC score alone. The machine learning-based risk prediction algorithm and the conventional CVD risk prediction models were compared in their ability to categorize patients in low-, intermediate-, or high-risk categories in terms of all-cause mortality. The CAC score was as good as the Framingham risk score in classifying patients as low risk for all-cause mortality but better than the Framingham risk score in distinguishing high-risk from intermediate-risk patients. The CAC score was also better than the ASCVD risk score in identifying patients who had a higher risk for all-cause mortality.

Study limitations

Our systematic review yielded only four articles for inclusion, highlighting the paucity of studies on the CAC score among asymptomatic working-age adults. The most common reason for exclusion of studies was their focus on symptomatic patients, followed by older patients. It is logical that most CAC studies thus far have included symptomatic and/or older patients, as these are the population groups with the highest CVD prevalence. Many articles utilized data from the Multi-Ethnic Study of Atherosclerosis (MESA) study [33], the Heinz-Nixdorf Recall study [34], and the Kangbuk Samsung Health Study [35]. These three studies included participants with ages outside the scope of the current investigation (i.e., adults older than 65 years of age), leading to the exclusion of resultant papers from our systematic review. In addition, articles published before November 2009 were also excluded. Further limitations of our systematic review include the fact that only four articles met the inclusion criteria; therefore, the generalizability of the results is limited. Additionally, the heterogeneity of the studies allowed only a qualitative assessment, preventing us from performing a quantitative meta-analysis. Because of the heterogeneity of the studies, only a limited comparison of baseline characteristics could be performed, and that prevented us from performing a quantitative meta-analysis with adjustment for confounding variables. Furthermore, additional potential limitations may refer to the differences in study designs, populations, and methodologies of included studies that may explain variations in findings. Specifically, the included studies were different with respect to variability in follow-up durations and differing CAC thresholds for risk classification. Finally, limitations in the study selection were the exclusion of non-English language studies, a single-database reliance, and the lack of consideration for grey literature.

Future research directions

Prospective studies are warranted to perform cost-benefit analyses on the utility of the CAC score to identify those age-, gender-, and CVD risk-specific subsets of the population that would benefit the most from screening. Cost-benefit analyses could include specific parameters, such as healthcare cost savings, adherence to preventive therapies, and impact on mortality.

## Conclusions

Our review highlights a knowledge gap in the literature that could be bridged with further studies, particularly focusing on asymptomatic working-age individuals. The knowledge gap could also be addressed by age-restricted reanalysis of larger existing datasets (e.g., MESA and CARDIA) from asymptomatic individuals. Future research could also focus on long-term outcomes, cost-effectiveness, and the impact of CAC scoring on clinical decision-making.

Our study demonstrated a higher risk of incident cardiac events and mortality among asymptomatic working-age adults with a CAC score greater than zero. In addition, increasing CAC score is associated with a higher risk of incident cardiac events and/or all-cause mortality in a dose-response relationship, indicating a possible criterion of causality in combination with the strength of association as evidenced by the specific, relatively large hazard ratios (four- to fivefold increased risk) from the studies. Furthermore, the CAC score has a better discriminatory power compared to the Framingham risk score and the ASCVD score in distinguishing high-risk from intermediate-risk individuals for all-cause mortality. The above findings suggest a measurable benefit of using the CAC score as a screening tool among asymptomatic working-age adults.
